# Structural analysis of a ligand-triggered intermolecular disulfide switch in a major latex protein from opium poppy

**DOI:** 10.1107/S2059798324007733

**Published:** 2024-08-29

**Authors:** Samuel C. Carr, Peter J. Facchini, Kenneth K. S. Ng

**Affiliations:** a Department of Biological Sciences University of Calgary Calgary AlbertaT2N 1N4 Canada; b Department of Chemistry and Biochemistry University of Windsor Windsor OntarioN9B 3P4 Canada; University of Queensland, Australia

**Keywords:** opium poppy latex, X-ray crystallography, ligand-coupled conformational changes, protein structure and function, plant specialized metabolism, benzylisoquinoline alkaloid biosynthesis

## Abstract

Several proteins from plant pathogenesis-related family 10 (PR10) are highly abundant in the latex of opium poppy and have recently been shown to play diverse and important roles in the biosynthesis of benzylisoquinoline alkaloids (BIAs). The recent determination of the first crystal structures of PR10-10 showed how large conformational changes in a surface loop and adjacent β-strand are coupled to the binding of BIA compounds to the central hydrophobic binding pocket. A more detailed analysis of these conformational changes is now reported to further clarify how ligand binding is coupled to the formation and cleavage of an intermolecular disulfide bond that is only sterically allowed when the BIA binding pocket is empty. To decouple ligand binding from disulfide-bond formation, each of the two highly conserved cysteine residues (Cys59 and Cys155) in PR10-10 was replaced with serine using site-directed mutagenesis. Crystal structures of the Cys59Ser mutant were determined in the presence of papaverine and in the absence of exogenous BIA compounds. A crystal structure of the Cys155Ser mutant was also determined in the absence of exogenous BIA compounds. All three of these crystal structures reveal conformations similar to that of wild-type PR10-10 with bound BIA compounds. In the absence of exogenous BIA compounds, the Cys59Ser and Cys155Ser mutants appear to bind an unidentified ligand or mixture of ligands that was presumably introduced during expression of the proteins in *Escherichia coli*. The analysis of conformational changes triggered by the binding of BIA compounds suggests a molecular mechanism coupling ligand binding to the disruption of an intermolecular disulfide bond. This mechanism may be involved in the regulation of biosynthetic reactions in plants and possibly other organisms.

## Abstract 2

Crystal structures of two cysteine-to-serine mutants of PR10-10 help to reveal how a ligand-induced conformational change is coupled to the formation of intermolecular disulfide bonds in plant pathogenesis-related family 10 proteins involved in the biosynthesis of benzylisoquinoline alkaloids.

## 1. Introduction

Disulfide bonds play diverse biological roles that can dramatically affect the folding, stability and function of proteins (Thornton, 1981 ▸). Defects in the formation of disulfide bonds can lead to deleterious effects associated with disease (Harper & Lansbury, 1997 ▸), and the intentional manipulation of disulfide-bond formation *in vitro* and *in vivo* has led to a deeper understanding of the natural functions of disulfide bonds, as well as their potential for applications in biotechnology and medicine. The formation of a disulfide bond through the oxidation of two cysteine thiol groups, and the cleavage of the covalent disulfide linkage by reduction, are readily reversible reactions under physiological conditions. *In vivo*, oxidation and reduction reactions involving disulfide bonds are typically catalyzed by a diversity of oxidoreductase enzymes, protein chaperones and small molecules. *In vitro*, oxygen can oxidize free thiols to from disulfide bonds, while reductants such as dithiothreitol (DTT) can cleave disulfide bonds into free thiols. Disulfide bonds have also been proposed to be cleaved through hydrolysis in the absence of reducing agents (Hogg, 2003 ▸; Churchfield *et al.*, 2016 ▸).

Although many disulfide bonds in proteins play a primarily static and structural role to stabilize the folded state of the protein, there is a growing awareness that the reversible formation and cleavage of some disulfide bonds can be triggered by dynamic physiological processes to regulate conformational changes that in turn control the functions of proteins (Hogg, 2003 ▸; Chiu & Hogg, 2019 ▸). The structural and functional effects of these so-called allosteric disulfides have been thoroughly studied in over 40 proteins, and it is likely that allosteric disulfides also play important roles in regulating the structure and function of many other proteins.

Recent work from our group suggested for the first time that the formation and cleavage of an intermolecular disulfide bond in plant pathogenenesis-related family 10 (PR10) proteins may be linked to the binding and storage of benzyl­isoquinoline alkaloids (BIAs) in the opium poppy *Papaver somniferum* (Ozber *et al.*, 2022 ▸). BIAs are a diverse family of plant specialized metabolites that are primarily found in the order Ranunculales, which includes important pharmaceutical compounds such as noscapine (a tumor-growth inhibitor), papaverine (a vasodilator) and morphine (an analgesic) (Dastmalchi, Park *et al.*, 2018 ▸). Very recently, our group discovered that major latex proteins (MLPs) and PR10 proteins play functionally important roles in the binding and storage of BIAs in opium poppy (Ozber *et al.*, 2022 ▸). MLP/PR10 proteins belong to the pathogenesis-related (PR) protein superfamily together with 16 other families classified based on overall sequence and structural similarity (van Loon & van Strien, 1999 ▸; van Loon *et al.*, 2006 ▸). Although the original naming of PR proteins derives from their initial discovery as proteins expressed in diseased tobacco leaves (van Loon & van Kammen, 1970 ▸), subsequent work has shown that PR10 proteins play diverse biological roles in defense against fungal (Chadha & Das, 2006 ▸), viral (Park *et al.*, 2004 ▸) and nematode (Andrade *et al.*, 2010 ▸) pathogens, as well as in cyanogenesis in herbivore defense (Lanfranchi *et al.*, 2017 ▸), cryoprotection (Ukaji *et al.*, 2004 ▸) and catalytic roles in plant specialized metabolism (Morris *et al.*, 2021 ▸). Central to the structure and function of all PR10 proteins is the conservation of the ancestral Bet v 1 fold, which harbors a hydrophobic binding pocket (Radauer *et al.*, 2008 ▸). Many PR10 proteins have been shown to have substantial affinity for hydrophobic ligands, including a diverse range of intermediates in the complex BIA biosynthetic pathways (Morris *et al.*, 2021 ▸). Additionally, three PR10 proteins catalyze diverse enzymatic reactions in BIA biosynthesis: the Pictet–Spengler acid-catalyzed condensation of amino and carbonyl groups followed by the ring-closure reaction catalyzed by norcoclaurine synthase (NCS; Samanani *et al.*, 2004 ▸), the allylic rearrangement reaction catalyzed by thebaine synthase (THS; Chen *et al.*, 2018 ▸) and the tautomerization reaction catalyzed by neopinone isomerase (NISO; Dastmalchi *et al.*, 2019 ▸). In addition to the catalytic activity of NCS, THS and NISO, however, many other MLP/PR10 proteins with no known functions are found at high levels in the soluble proteome of opium poppy latex (Dastmalchi *et al.*, 2019 ▸), and we recently showed that many of these proteins also bind to a broad range of BIA ligands and co-sediment with alkaloids and BIA biosynthetic enzymes in high-density sucrose gradient fractions (Ozber *et al.*, 2022 ▸). These findings suggest that protein bodies or biomolecular condensates formed by the aggregation of proteins and alkaloids may facilitate the accumulation of highly concentrated BIAs in cellular subcompartments that are critical for the efficient biosynthesis of a diversity of BIA compounds (Ozber *et al.*, 2022 ▸).

To understand the molecular basis of alkaloid binding to PR10 proteins, we determined structures of PR10-10 using X-ray crystallography (Ozber *et al.*, 2022 ▸). We observed that an intermolecular disulfide bond between Cys59 and Cys155 is formed in the apo structure, while the binding of alkaloid compounds to the central hydrophobic pocket induces conformational changes that prevent the formation of this intermolecular disulfide bond. These observations suggest that the binding of ligands to PR10-10 is coupled to the reduction and cleavage of an intermolecular disulfide bond, which is in turn linked to the disruption of intermolecular interactions needed to form oligomeric assemblies that may have important functional roles in the biosynthesis of BIA compounds. In other *P. somniferum* PR10 proteins, the high level of sequence conservation of the disulfide-bond-forming Cys59 and Cys155 alongside another surface cysteine, Cys21, suggests that the structure–function relationships observed in PR10-10 reflect a more general mechanism for functional disulfides that may exist in many other PR10 proteins.

To further explore the molecular basis of disulfide-mediated oligomerization and alkaloid ligand-induced disulfide-bond cleavage in PR10-10, we have investigated the structural and functional effects of mutations of the highly conserved cysteine residues. Although efforts were made to crystallize the cysteine mutants in the absence of ligands, the resulting crystal structures showed electron density for unknown ligands in the active site that were presumably derived from the bacterial host used for recombinant protein expression. These structures are nearly identical to the crystal structure of the Cys59Ser mutant cocrystallized with papaverine and the previously reported structures that we determined for PR10-10 in complex with different BIA compounds. Structural analysis of the structures determined for both mutant proteins in combination with a deeper analysis of the previously published wild-type PR10-10 crystal structures reveals additional features of the molecular mechanisms underlying the disulfide switch that regulates PR10-10 oligomerization in response to BIA binding.

## 2. Materials and methods

### 2.1. Chemicals

Crystallization screens and reagents were purchased from Hampton Research. Papaverine and media components were purchased from Sigma–Aldrich. Controlled substances were acquired and used under a Canadian federal governmental license.

### 2.2. Site-directed mutagenesis

Site-directed mutagenesis was performed on *P. somniferum* PR10-10 pET-47b plasmid (Ozber *et al.*, 2022 ▸) using previously described methods (Zheng *et al.*, 2004 ▸; Dastmalchi, Chang *et al.*, 2018 ▸). Cysteine-to-serine substitutions at positions 21, 59 and 155 were introduced by PCR-mediated site-directed mutagenesis using Q5 High-Fidelity DNA polymerase (New England Biolabs) and oligonucleotide primers with the desired nucleotide substitutions (Integrated DNA Technologies; Supplementary Table S1). The corresponding pET-47b PR10-10 constructs were verified using dideoxynucleotide chain-terminator sequencing (Supplementary Table S2).

### 2.3. Protein expression and purification

The pET-47b expression constructs for the Cys21Ser, Cys59Ser and Cys155Ser mutants of PR10-10 were transformed into *Escherichia coli* ArcticExpress (DE3) competent cells (Agilent Technologies). Starter cultures were inoculated with transformed cells and grown at 25°C with shaking at 170 rev min^−1^ overnight in 50 ml Luria–Bertani (Miller) broth supplemented with 30 mg l^−1^ kanamycin and 45 mg l^−1^ chloramphenicol (LBKC) to an OD_595_ of ∼0.4, and were subsequently used to inoculate two 1 l cultures per cysteine mutant in LBKC. The cultures were grown at 30°C until the OD_595_ reached 0.4–0.6, followed by cooling at 16°C for 30 min. Recombinant protein expression was induced by the addition of isopropyl β-d-1-thiogalactopyranoside to a final concentration of 1 m*M*, and the cultures were incubated at 16°C for 24 h with shaking at 170 rev min^−1^. The cells were harvested by centrifugation and resuspended in lysis buffer [50 m*M* sodium phosphate pH 8.0, 300 m*M* sodium chloride, 15%(*v*/*v*) glycerol]. The resuspended pellets were stored at −80°C until thawed for subsequent lysis and protein purification. The thawed resuspended pellets were lysed by sonication in the presence of lysozyme, DNase and phenylmethylsulfonyl fluoride. Soluble proteins were separated from cell debris by centrifugation at 4°C. The supernatants were incubated with 1 ml TALON resin (Clontech) equilibrated with lysis buffer for 45 min on ice with shaking at 65 rev min^−1^. The resin was subsequently washed with 10 ml lysis buffer and incubated in 40 ml lysis buffer for 45 min with shaking at 65 rev min^−1^ on ice. The resin was then washed with 20 ml lysis buffer supplemented with 5 m*M* imidazole followed by another 2 ml lysis buffer supplemented with 10 m*M* imidazole. The recombinant proteins were eluted with 4 ml lysis buffer supplemented with 200 m*M* imidazole at 1 ml intervals. The imidazole concentration was decreased to less than 1 m*M* in proteolysis buffer (50 m*M* bis-Tris–HCl pH 7.0, 150 m*M* NaCl, 1 m*M* EDTA, 1 m*M* DTT, degassed water) by ultrafiltration (10K), followed by overnight digestion using PreScission protease (ThermoFisher, USA) to cleave the 6×His tag. Glutathione *S*-transferase-tagged protease was removed by running the digested protein through glutathione Sepharose 4B resin (GE Healthcare, USA). The cleaved proteins were dialyzed overnight against the final buffer (20 m*M* Tris–HCl pH 8.0, 30 m*M* NaCl) and spin-concentrated (10K) to a final concentration of 12–15 mg ml^−1^. Protein purity was determined by SDS–PAGE (Supplementary Fig. S1) and the protein concentration was determined from the absorbance at 280 nm based on the extinction coefficient calculated from the amino-acid composition (Gill & von Hippel, 1989 ▸). Concentrated proteins were flash-frozen in liquid nitrogen and stored at −80°C.

### 2.4. Crystallization and X-ray crystallography

All crystallizations were performed using the hanging-drop vapor-diffusion method at room temperature. The PR10-10-Cys59Ser–papaverine complex was crystallized in the presence of 1 m*M* papaverine (approximately 1:1 molar ratio of protein to BIA) and 10%(*v*/*v*) methanol using 17%(*w*/*v*) polyethylene glycol 3350, 0.1 *M* bis-Tris pH 5.1 as the well solution. PR10-10-Cys59Ser was crystallized in the absence of exogenously added BIA ligands using 18% polyethylene glycol 3350, 0.1 *M* bis-Tris pH 5.5, 0.2 *M* sodium chloride as the well solution. PR10-10-Cys155Ser was crystallized in the absence of exogenously added BIA ligands using 23%(*w*/*v*) polyethylene glycol 3350, 0.1 *M* bis-Tris pH 5.5, 0.2 *M* sodium chloride. The Cys59Ser and Cys155Ser mutants were crystallized using the same conditions as the previously published apo PR10-10 structure (Ozber *et al.*, 2022 ▸; no addition of sodium chloride); however, the addition of sodium chloride resulted in crystals with slightly higher resolution X-ray diffraction. Crystallization drops were coated with perfluoro­polyether cryogenic oil (Hampton Research, USA) prior to harvesting and flash-cooling. Single crystals were harvested using polymer loops (MiTeGen, USA), flash-cooled in liquid nitrogen and stored in liquid nitrogen before data collection. X-ray diffraction data were obtained on beamline 12-2 at the Stanford Synchrotron Radiation Laboratory (SSRL) using radiation at a wavelength of 0.98 Å and a PILATUS 6M pixel array detector (Dectris, Switzerland). Crystals were cooled under a nitrogen gas stream at 100 K during data collection. *XDS* and *XSCALE* (Kabsch, 2010 ▸) were used for data processing, and the PR10-10 cysteine-mutant crystal structures were solved by molecular replacement using the crystal structure of wild-type PR10-10 with bound papaverine (PDB entry 7uqm; Ozber *et al.*, 2022 ▸) as the search model with *Phaser*, as implemented in *Phenix* (Liebschner *et al.*, 2019 ▸). Refinements were conducted with *Phenix*, *Coot* (Emsley *et al.*, 2010 ▸) was used for model building, and model quality was assessed using *MolProbity* (Davis *et al.*, 2007 ▸). The coordinates for papaverine were obtained from the Cambridge Structural Database (CSD; Refcode MVERIQ01), and *ProDRG* (Schüttelkopf & van Aalten, 2004 ▸) was used to generate restraint files for refinement. Structure coordinates were deposited in the PDB for PR10-10-Cys155Ser (PDB entry 8vo1), PR10-10-Cys59Ser (PDB entry 8vo2) and PR10-10-Cys59Ser–papaverine (PDB entry 8vo3).

### 2.5. Structural analyses

*PyMOL* (Schrödinger) was used for structure visualization and solvent-accessible surface calculations. The *SPACEBALL* server version 2.0 was used for internal cavity calculations with a probe radius of 1.42 Å, a lattice constant of 0.6 Å and five rotations (Chwastyk *et al.*, 2014 ▸, 2016 ▸). *Phenix* was used to calculate both simulated-annealing omit maps and polder maps (Liebschner *et al.*, 2017 ▸, 2019 ▸).

## 3. Results

### 3.1. Overall structure of PR10-10 cysteine mutants

**Figure 1:**
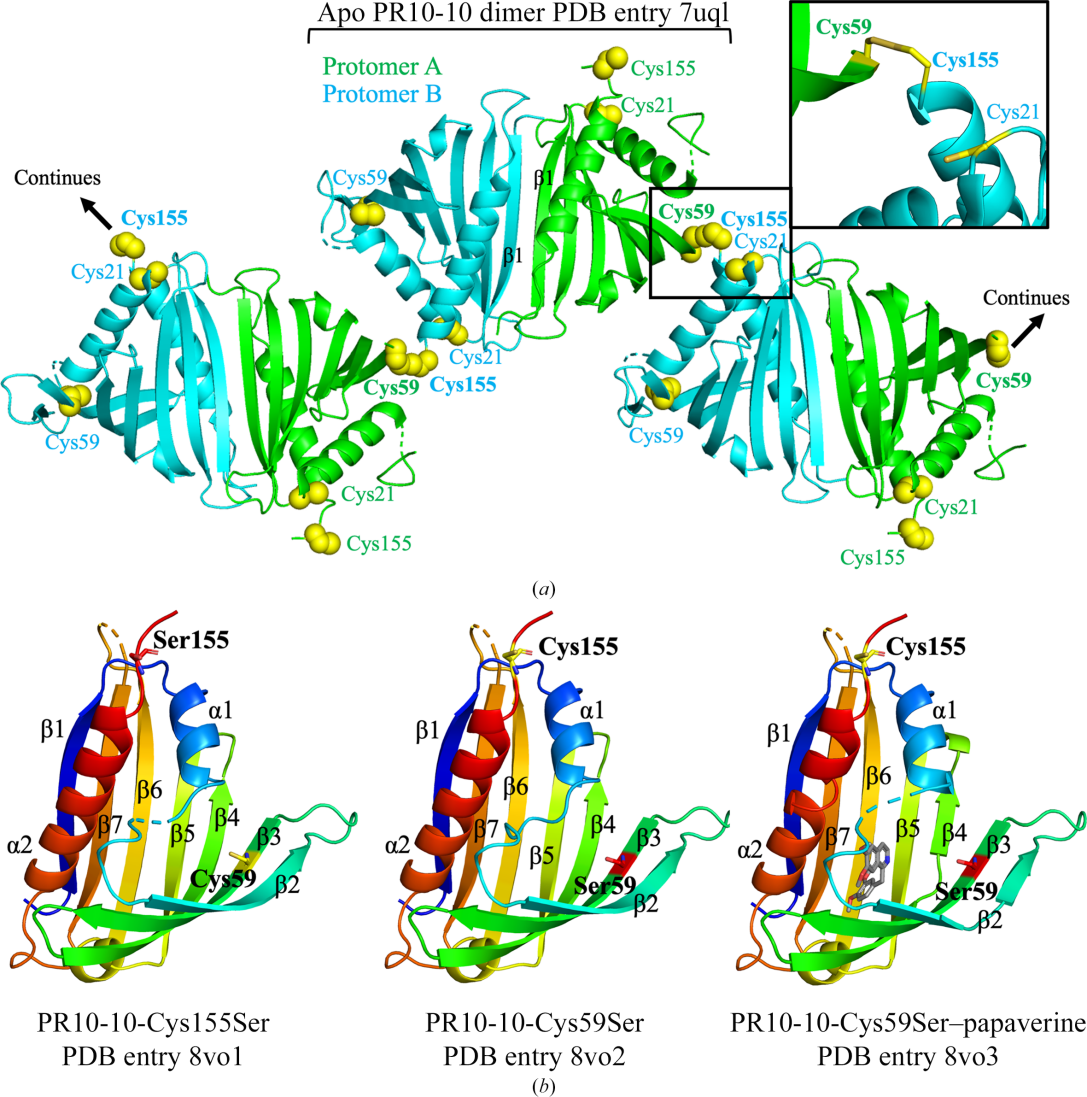
PR10-10 cysteine-mutant crystal structures. (*a*) Disulfide-mediated linkage of PR10-10 homodimers. The conserved PR10-10 homodimer is indicated with protomers shown in green and cyan, and the β1 strands forming the center of the dimer interface are labeled. Conserved cysteines 21, 59 and 155 are shown in yellow and the disulfide-forming residues are shown in bold. Additionally, the continuation of the in-crystal polymer is designated. (*b*) PR10-10-Cys155Ser and PR10-10-Cys59Ser crystal structures determined in the absence of exogenous BIA ligands with bound unidentified ligands that were presumably copurified from *E. coli*, and the crystal structure of PR10-10-Cys59Ser with bound papaverine. Papaverine is shown in gray, whereas the unidentified ligands were not modeled. The key residues Cys59, Cys155, Ser59 and Ser155 and secondary-structural elements are labeled. The rainbow coloring scheme starts with the N-terminus in blue and ends with the C-terminus in red.

**Figure 2:**
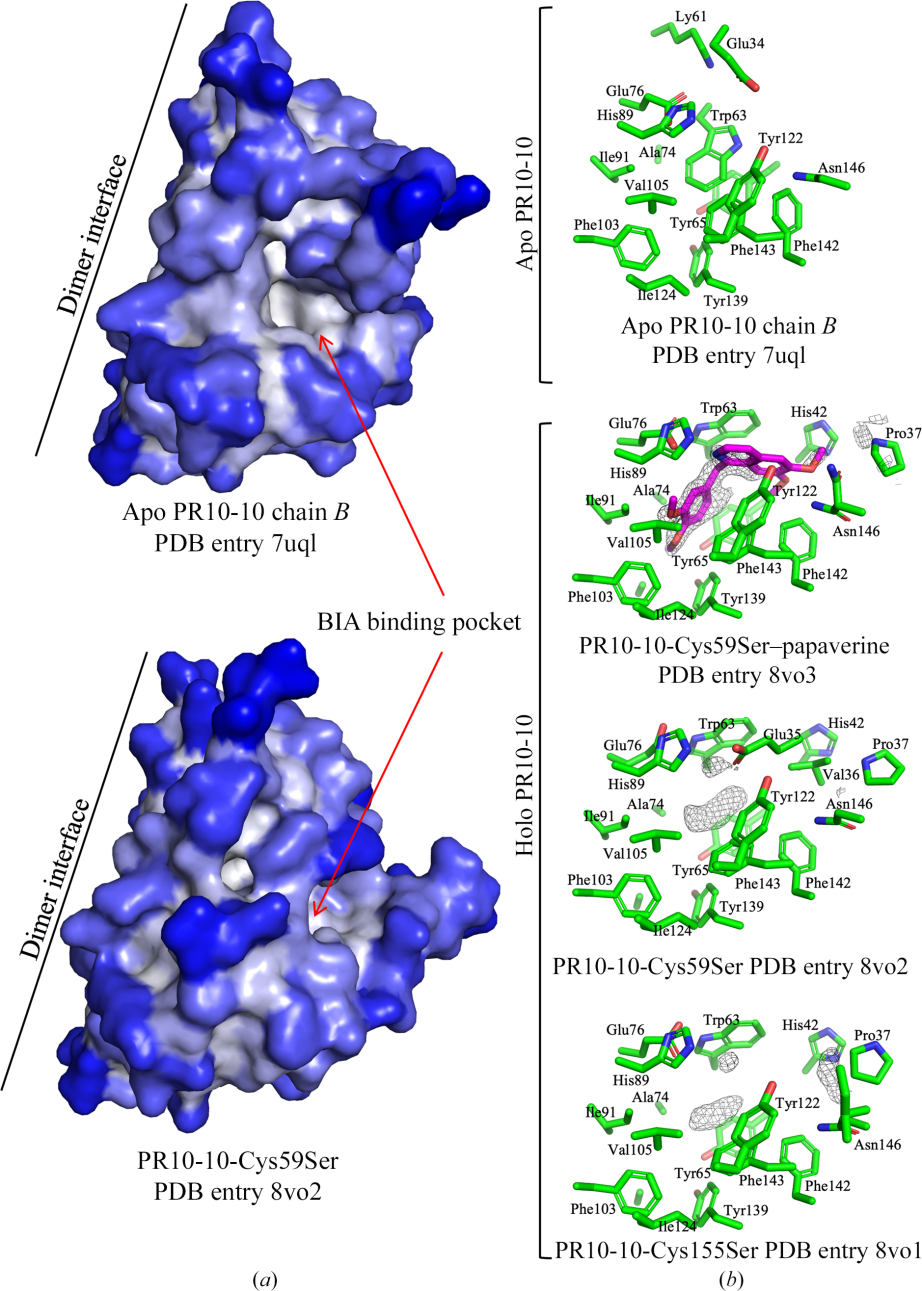
The BIA binding pocket in apo and holo PR10-10. (*a*) Solvent-accessible surface depiction of wild-type PR10-10 and PR10-10-Cys59Ser. Coloring corresponds to solvent accessibility, with white being the least accessible and blue being the most accessible. Individual PR10-10 protomers are shown with the dimer interface marked. The BIA binding pocket is also marked by red arrows. (*b*) Close-up views of the binding pockets of the apo and holo structures of wild-type PR10-10 and the two cysteine mutants. The simulated-annealing (|*F*_o_| − |*F*_c_|) omit map is contoured at 3σ and is represented as a gray mesh. The starting and ending temperatures for simulated annealing were 5000 and 300 K, respectively, using *Phenix* (Liebschner *et al.*, 2019 ▸). Protein C atoms and bonds are shown in green, ligand C atoms and bonds in magenta, O atoms in red and N atoms in blue. An additional polder map for PR10-10-Cys59Ser–papaverine is shown in Supplementary Fig. S2.

The holo crystal structures of PR10-10 cysteine mutants Cys59Ser and Cys155Ser were both solved in complex with an unknown ligand or, more likely, a mixture of unknown ligands. In the case of the Cys59Ser mutant, a structure was also determined in complex with papaverine. Despite crystallization trials, the Cys21Ser mutant failed to yield well diffracting crystals. Structures were solved by the molecular-replacement method using the crystal structure of wild-type PR10-10 with bound papaverine (PDB entry 7uqm) as a search model. The structures of the Cys59Ser mutant were refined to 1.5 Å resolution and the structure of the Cys155Ser mutant was refined to 1.8 Å resolution (Table 1 ▸). Each of these complexes crystallized isomorphously with the previously reported wild-type PR10-10 complexes. A single copy of the protein is found in the asymmetric unit of a crystal form with *C*222_1_ space-group symmetry. A dimer with an intermolecular interface centered on the α2 helix is generated by a twofold crystallographic symmetry rotation axis (Fig. 1 ▸*a*). As expected, both PR10-10 cysteine-mutant structures show the same overall Bet v 1 fold as seen in wild-type PR10-10, THS and other members of the PR10 family (Fernandes *et al.*, 2013 ▸), in which seven antiparallel β-strands cradle a central α2 helix (Fig. 1 ▸*b*). The BIA binding pocket is located in a central hydrophobic cleft within the β-strand flanked by the central α2 helix and auxiliary α1 helix. In holo PR10-10, the BIA binding pocket is partially excluded from the solvent by the ordering of a loop, dubbed the cap loop (residues 32–42), formed by partial unraveling of the α1 helix. Insufficient electron density was present to model Glu34, Glu35 and Val36 in PR10-10-Cys59Ser–papaverine and PR10-10-Cys155Ser. However, the higher quality of the electron density in this region allowed the modeling of the whole cap loop in PR10-10-Cys59Ser with a bound unknown ligand. Similar to the PR10-10-Cys59Ser–papaverine and apo PR10-10 structures, previous structures of wild-type PR10-10 also showed insufficient electron density to model the entire cap loop. This region of weak electron density is proximal to the bound papaverine. Based on papaverine omit maps, it appears that the benzyl moiety of papaverine is well anchored in the binding pocket, while the isoquinoline moiety extends into the more disordered region of the cap loop, also bearing a degree of disorder (Fig. 2 ▸ and Supplementary Fig. S2).

### 3.2. BIA binding triggers conformational changes in the cap-loop region of PR10 proteins

**Figure 3:**
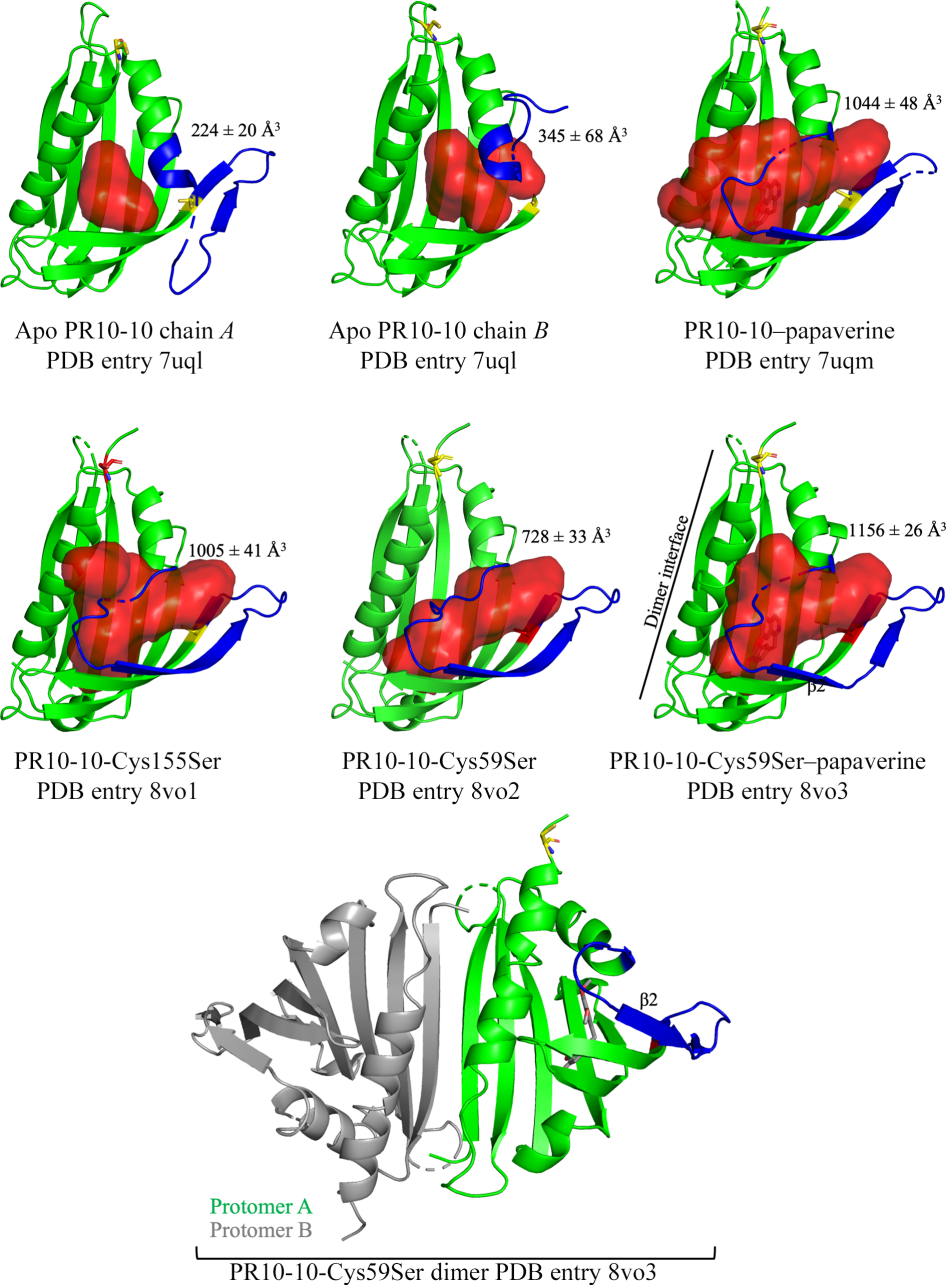
PR10-10 cap-loop conformations. The cap-loop conformations in PR10-10 crystal structures and their binding pockets. The cap loops and β2 strands are highlighted in blue. Calculated largest internal cavities (*i.e.* binding pockets) are shown in red and labeled with their volumes (Chwastyk *et al.*, 2014 ▸, 2016 ▸). Cysteine residues are shown in stick format and highlighted in yellow, whereas the corresponding Cys→Ser mutations are highlighted in red. The conserved PR10-10 homodimer is shown at the bottom with the displayed protomer in green and its partner in gray. The single protomers and the dimer have slightly different orientations in order to better showcase their features. The dimer interface along the β1 strand is indicated by a black line.

The binding of ligands to PR10-10 induces large conformational changes that have not been previously analyzed or reported in detail. To provide a framework for understanding the effects of the cysteine-to-serine mutations at positions 59 and 155 of PR10-10, we conducted a detailed analysis of our previously published apo PR10-10 and three BIA-complexed holo PR10-10 structures (Ozber *et al.*, 2022 ▸), highlighting in particular the series of conformational changes to the cap-loop and β2-strand region occurring in response to BIA binding (Fig. 3 ▸). Binding of papaverine (PDB entry 7uqm), (*S*)-tetrahydropapaverine (PDB entry 7uqo) and noscapine (PDB entry 7uqn) appears to induce a shift of the cap loop (residues 32–42) into a closed state and ordering of the β2 strand (residues 42–50), allowing the cap loop and β2 strand to form a lid over the BIA binding pocket that helps to shield hydrophobic parts of the bound BIA compound from the solvent. In contrast, our previously published wild-type apo PR10-10 crystal structure (PDB entry 7uql) reveals the presence of distinct conformations corresponding to open states of the cap loop and varying disorder of the β2 strand in the two different protomers found in the asymmetric unit (Fig. 3 ▸). The conformation adopted by chain *A* reveals an open cap loop and a partially ordered β2 strand. The partially ordered β2 strand consists of residues 47–50, forming antiparallel β-sheet pairing interactions with the β3 strand that are similar to those seen in the BIA-bound structures of PR10-10. In contrast, in chain *B* the β2 strand is fully disordered, which importantly exposes Cys59 to the solvent. Residues 41–49 adopt a conformation interacting with the outside of the α1 helix. In both chains *A* and *B* residues 37–40, corresponding to the C-terminal end of the cap loop, are disordered. In both cases the BIA binding pocket is exposed to the solvent. The distinct conformations observed in chains *A* and *B* of apo PR10-10 both suggest how conformational changes in the cap loop and the N-terminal part of the β2 strand combine to expose the hydrophobic BIA binding pocket to solvent for the entry of BIA compounds. In the crystal structure of apo PR10-10, the open conformations adopted by the cap loops in both copies appear to be partially stabilized by crystallo­graphic packing interactions.

In contrast, the binding of papaverine, (*S*)-tetrahydro­papaverine and noscapine to the BIA binding pocket reveal a closed conformation of the cap loop that shields the bound BIA compound from the solvent. Residues 33–36 in the α1 helix unwind and adopt a nonhelical backbone conformation to close off the BIA binding pocket. Our new crystal structures, from which the full cap loop is modeled, reveal that the side chain of Glu35 points into the BIA binding pocket after the C-terminal end of the α1 helix (Figs. 2 ▸ and 4 ▸). The shifting of the cap loop over the BIA binding pocket is subsequently accompanied by ordering of the β2 strand, which forms an antiparallel β-sheet with the β3 strand. The BIA binding pocket is mostly unchanged in the apo and holo states, except for the side chain of Trp63, which in the apo conformation points into the binding pocket, thereby occupying most of the hydrophobic cleft. BIA binding causes the indole side chain of Trp63 to move out of the binding pocket and into a flanking position, which likely contributes to aromatic interactions with the bound BIA. Analysis of the internal cavities (*i.e.* binding pockets) of the various PR10-10 structures reveal a smaller cavity for the open conformations of apo PR10-10 compared with the holo conformations (Fig. 3 ▸). The smaller cavity in apo PR10-10 is defined by the ordering of residues 33–36, extending the α1 helix into the binding pocket, and the rotation of the Trp63 side chain into the binding pocket. The unraveling of the α1 helix and shifting of the Trp63 side chain in holo PR10-10 functionally widen the binding pocket, making space for the bound ligand. Despite the same crystallization conditions, apo and holo PR10-10 adopt crystal forms with distinct space groups, reflecting different crystal-packing arrangements caused by the differences in the two conformational states. Apo PR10-10 crystals belong to space group *P*2_1_, whereas holo PR10-10 crystals belong to space group *C*222_1_. The space-group symmetry and the conformation of the Trp63 side chain are diagnostic characteristics of apo and holo PR10-10. Interestingly, and relevant to protein aggregation in response to BIA binding, ordering of the β2 strand buries Cys59, thus preventing the side-chain thiol group from forming an intermolecular disulfide bond with Cys155 as observed in the apo PR10-10 crystal structure. Because the formation of this intermolecular disulfide bond is a central part of the intermolecular interface formed by the head-to-tail in-crystal polymer, we hypothesize that BIA binding and release could trigger the oligomerization and depolymerization of PR10-10. To test this hypothesis, we expressed, purified and crystallized the PR10-10 Cys59Ser and Cys155Ser mutants to assess the role of intermolecular disulfide-bond formation in protein oligomerization and BIA binding. In addition to Cys59 and Cys155, Cys21 is also conserved in *P. somniferum* PR10s (Ozber *et al.*, 2022 ▸). Interestingly, Cys21 is located near the surface and in proximity to Cys155, with the α-carbons being within 8 Å of each other (Fig. 1 ▸*a*). Although not observed in the crystal structures, slight conformational changes to Cys21 and Cys155 may facilitate the formation of an intra­molecular disulfide bond, which in turn would impede the observed Cys59–Cys155 linkage. The conservation of Cys21 and its proximity to Cys155 and the protein surface makes it a good candidate for intermolecular and intramolecular disulfide-bond formation and was thus included in this study; however, as previously stated, structure determination of a Cys21 mutant was not successful.

### 3.3. Cysteine mutants and BIA binding trigger similar conformational modifications

**Figure 4:**
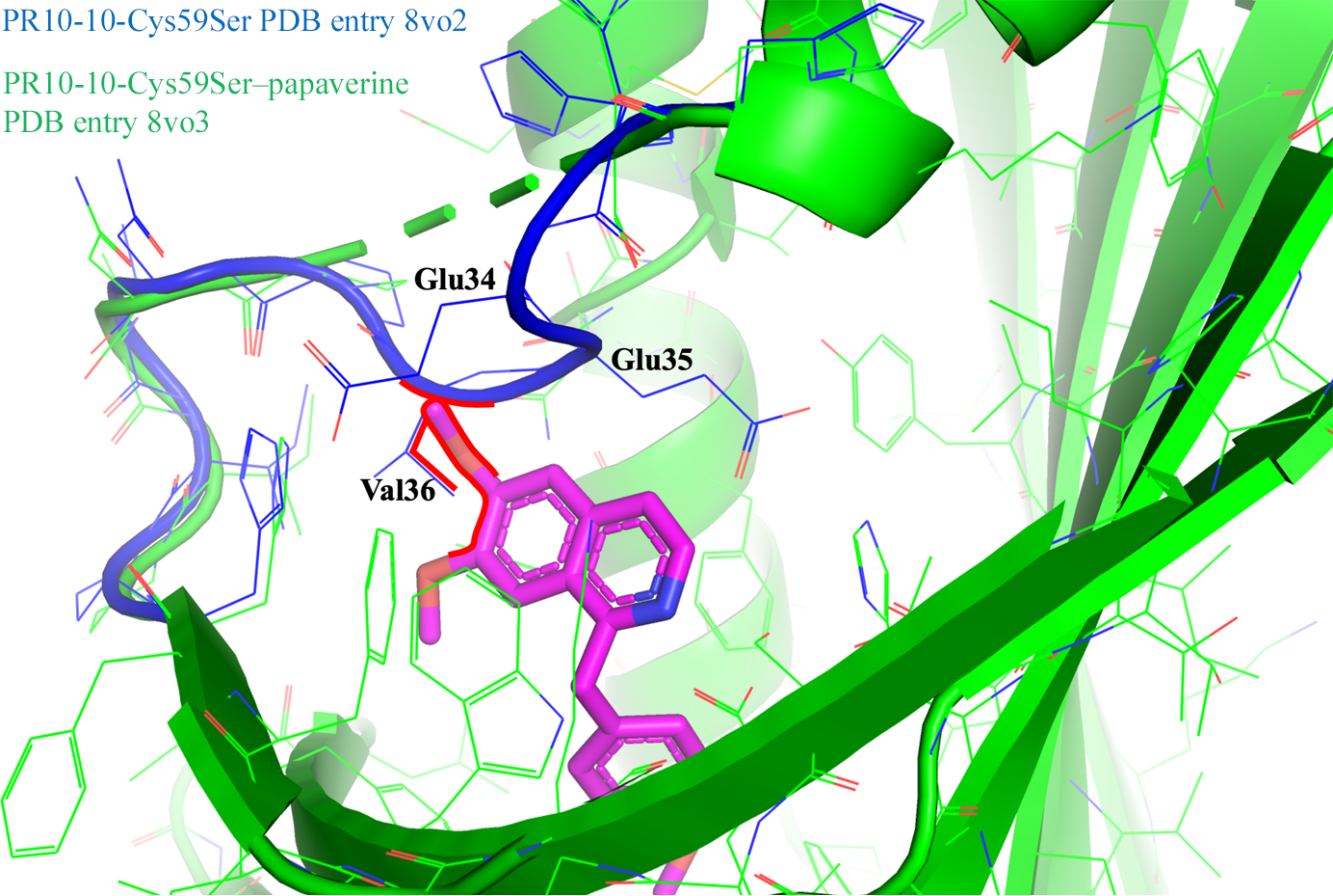
Comparison of the PR10-10-Cys59Ser cap loop in the papaverine-bound structure and the unknown ligand-bound structure. Structural superposition of PR10-10-Cys59Ser–papaverine and PR10-10-Cys59Ser with a bound unidentified ligand. Only the cap loop is shown for PR10-10-Cys59Ser–unidentified ligand. C atoms and bonds are shown in green (PR10-10-Cys59Ser–papaverine), blue (PR10-10-Cys59Ser–unidentified ligand) or magenta (papaverine). N atoms are shown in blue and O atoms in red.

Attempts to crystallize the Cys59Ser and Cys155Ser mutants of PR10-10 in the absence of BIA ligands surprisingly yielded crystals with the morphology characteristic of PR10-10 with bound BIA ligands. Data processing confirms that these crystals show *C*222_1_ space-group symmetry, and structure determination and refinement reveal a closed cap-loop conformation similar to the structures of PR10-10 with bound BIA ligands. Despite the absence of exogenously added ligands during crystallization, there appears to be electron density in the hydrophobic pocket corresponding to an unidentified compound (Fig. 2 ▸). Comparison of the PR10-10 Cys59Ser and Cys155Ser crystal structures with the wild-type PR10-10–papaverine complex (PDB entry 7uqm) demonstrates near-identical structures (Fig. 3 ▸). As expected, the conservative nature of the Cys59Ser and Cys155Ser substitutions does not appear to disrupt the overall structure of PR10-10. Although holo PR10-10 and cysteine-mutant PR10-10 structures all showed closed cap-loop conformations, and slight differences in the cap position from residues 37–40 were observed, it should be noted that residues 34–36 in wild-type PR10-10, residues 35–36 in PR10-10-Cys155Ser and residues 34–36 in PR10-10-Cys59Ser–papaverine were not sufficiently well ordered to be modeled. The PR10-10-Cys59Ser and PR10-10-Cys155Ser structures demonstrated a similar cap-loop conformation, which was fully modeled for PR10-10-Cys59Ser. Residues 38–40 were shifted ∼3.8 Å perpendicularly away from the central α2 helix in the PR10-10 cysteine mutants compared with wild-type holo PR10-10. The key cap-loop residues Glu35 and Val36, which contribute to the BIA binding pocket in the holo form, are shifted ∼4 Å into the BIA binding pocket in PR10-10-Cys59Ser compared with wild-type holo PR10-10, thereby decreasing the size of the pocket. Structural alignments showed that the smaller BIA binding pocket in PR10-10-Cys59Ser does not provide sufficient space for papaverine binding, specifically the position of main and side chain of Val36 (Fig. 4 ▸). The cap loop appears to be capable of adopting different conformations to accommodate the binding of different-sized ligands. This suggests that cap-loop flexibility increases the scope of available PR10-10 ligands, highlighting a potentially important evolutionary characteristic of MLP/PR10 proteins.

The open cap-loop conformation in apo PR10-10 appears to be locked open by the Cys59–Cys155 intermolecular disulfide bond. BIA binding and subsequent ordering of the cap loop into the closed conformation is not possible when the Cys59–Cys155 disulfide bond is formed. The binding of a high-affinity ligand is likely to be coupled to the reduction and breakage of the disulfide linkage. Mutation of either Cys59 or Cys155 was associated with the binding of an unknown molecule in the absence of an alkaloid ligand, whereas similar conditions for the purification, storage and crystallization of wild-type PR10-10 resulted in the apo conformation.

## 4. Discussion

BIA binding to PR10-10 has previously been shown to be coupled to the reduction of an intermolecular disulfide bond, allowing conformational changes that complete the alkaloid binding pocket and alter the oligomeric state of the protein (Ozber *et al.*, 2022 ▸). Crystal structures of PR10-10-Cys59Ser and PR10-10-Cys155Ser surprisingly show that replacing each cysteine residue with serine appears to disfavor the apo PR10-10 conformation. As a result, we were not able to crystallize the ligand-free state of either mutant protein. Instead, each mutant protein crystallized in a ligand-bound conformation in the absence of exogenous alkaloid, with the appearance of electron density in the ligand-binding site corresponding to an unknown small molecule that presumably copurified with each protein. The crystal structures reported in this paper and previous studies suggest the following: (i) in the absence of exogenous BIA ligands, wild-type PR10-10 adopts the apo conformation (PDB entry 7uql), (ii) the binding of BIA ligands to wild-type and mutant PR10-10 proteins induces a conformational change that promotes the reduction and cleavage of the intermolecular disulfide bond (PDB entries 7uqm, 7uqn, 7uqo and 8vo3), (iii) the Cys59Ser and Cys155Ser substitutions each preclude disulfide-bond formation and disfavor the adoption of apo conformations, which allows the binding of unknown ligands that are likely to be copurified from *E. coli* (PDB entries 8vo1 and 8vo2) and (iv) PR10-10-Cys59Ser shows preferential binding to papaverine over the non-BIA ligand (PDB entry 8vo3). Given the apparent coupling of the Cys59–Cys155 intermolecular disulfide bond and ligand binding, this disulfide bond may act as a biologically significant molecular switch.

**Figure 5:**
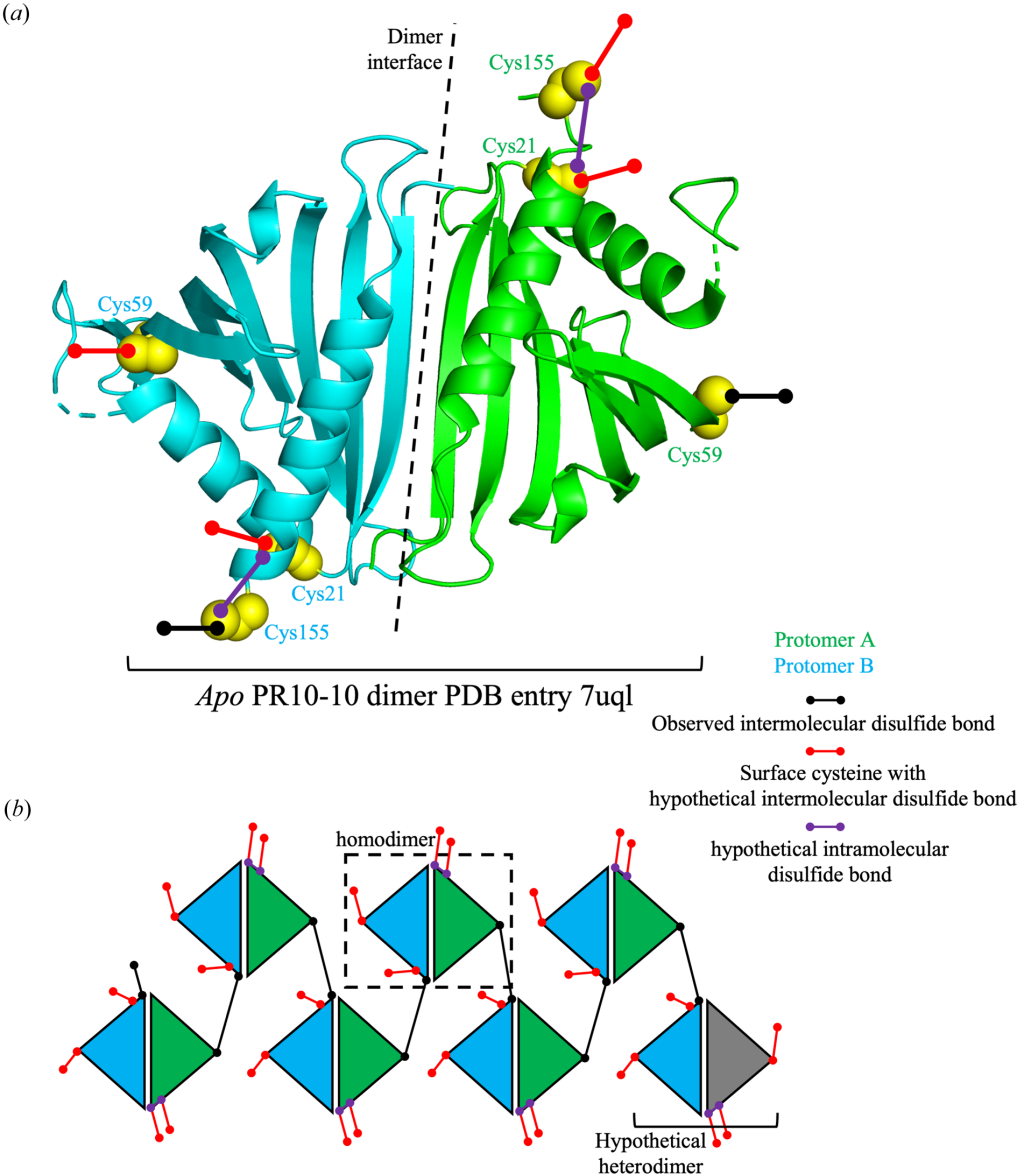
The conserved surface cysteines of PR10-10 and their possible linkages. (*a*) The PR10-10 homodimer as represented by the crystal structure of apo PR10-10 (PDB: 7uql) and its surface cysteines (Cys21, Cys59 and Cys155). (*b*) Diagram illustrating the observed disulfide bond mediated oligomerization of PR10-10 (black) and additional hypothetical disulfide bonds formed by other surface cysteines (red and purple). The hypothetical possibility of PR10 heterodimers is indicated using a grey protomer. PR10-10 protomers are shown in green and cyan.

PR10-10 has been proposed to act as a noncatalytic BIA binding protein (Ozber *et al.*, 2022 ▸). A shotgun proteomics approach was used to show that PR10-10 localizes to both the soluble and insoluble fractions in opium poppy latex (Dastmalchi *et al.*, 2019 ▸), whereas co-sedimentation studies with sucrose-gradient centrifugation suggest an association with alkaloids and BIA biosynthetic enzymes (Ozber *et al.*, 2022 ▸). Consistent with the binding and co-crystallization studies, as well as the proposed role in binding poorly soluble BIA compounds, PR10-10 was also shown to be most abundant in soluble latex fractions of the noscapine- and papaverine-accumulating opium poppy variety Roxanne (Dastmalchi *et al.*, 2019 ▸). These and other recent observations suggest a new model for alkaloid accumulation in opium poppy in which hydrophobic BIA compounds are stored in latex protein bodies or biomolecular condensate-like aggregations primarily formed by MLP/PR10 proteins (Ozber *et al.*, 2022 ▸). Within the context of this model, the co-regulation of the BIA-binding and oligomeric state of PR10-10 may represent a key mechanism in the formation and regulation of protein–alkaloid aggregates in the latex. The formation of intermolecular disulfide bonds by the homodimeric apo form of PR10-10 is expected to form complex networks in which each PR10-10 protomer can interact with adjacent protomers through four distinct contact points (Cys21, Cys59, Cys155 or the homodimer interface; Fig. 5 ▸). The crystal structure of apo PR10-10 (PDB entry 7uql) reveals a regularly repeating head-to-tail polymeric structure, but given the complex biological environment of the latex, it seems likely that PR10-10 oligomerization may also involve alternate disulfide linkages, other MLP/PR10 proteins (which have similar structures and conserved cysteine residues) and possibly various BIA biosynthetic enzymes. Given the observed Cys59–Cys155 linkage, each PR10-10 protomer has two additional free cysteines (Cys59/155 and Cys21) which may form additional disulfide bonds (Fig. 5 ▸). This raises the possibility of the formation of complex networks. The binding of a ligand to PR10-10 induces the ordering of the cap loop and disruption of the Cys59–Cys155 linkage by burying Cys59, thus dis­favouring or altering the formation of the predicted disulfide-linked network.

The proposed role of latex and alkaloids in plant defense against grazing herbivores, where BIA-containing latex is secreted from the plant (Schmeller *et al.*, 1997 ▸; Wink, 2003 ▸; Agrawal & Konno, 2009 ▸), provides an intriguing putative role for the redox-sensitivity of the disulfide molecular switch in sensing tissue damage through exposure to oxygen in the air. The overall structural conservation (Fernandes *et al.*, 2013 ▸) and the invariable occurrence of cysteine residues Cys21, Cys59 and Cys155 (PR10-10 numbering) among opium poppy MLP/PR10 proteins (Ozber *et al.*, 2022 ▸) suggest that other members of the MLP/PR10 family also have the potential to form similar disulfide-linked oligomers. It is interesting to note that disulfide bonds have been observed in the morphine biosynthetic enzymes codeinone reductase (Carr *et al.*, 2021 ▸) and salutaridine reductase (Higashi *et al.*, 2011 ▸), potentially indicating other partners for the formation of disulfide-linked molecular switches in opium poppy latex (Supplementary Fig. S3). Additionally, a similar in-crystal polymer of alternating disulfide bonds and β-sheet-mediated dimerization has been reported for the related PR protein Hyp-1 (Michalska *et al.*, 2010 ▸) and may represent a conserved feature in the protein family.

The dynamic formation and breakdown of disulfide-linked oligomeric networks may also be expected to involve regulation and catalysis by redox-active enzymes such as thio­redoxins (Nagahara *et al.*, 2007 ▸). Reservoirs of small-molecule oxidants and reductants may be involved (De Piña *et al.*, 2008 ▸). Interestingly, in mechanochemistry, shear force has been shown to alter the redox potential of disulfide bonds (Wiita *et al.*, 2006 ▸; Baldus & Gräter, 2012 ▸; Dopieralski *et al.*, 2013 ▸), which is pertinent given the large ligand-binding-induced conformational changes observed in PR10-10. The organization of disulfide-linked oligomeric networks within the proposed latex protein bodies or biomolecular condensates and the specificity of disulfide-bond formation are not known at present. The apparently spontaneous and reversible formation of the asymmetric Cys59–Cys155 disulfide bond in apo PR-10-10 *in vitro* during crystallization suggests the possible formation of symmetric Cys59–Cys59 and Cys155–Cys155 isomers, or the formation of disulfide bonds involving Cys21. *In vivo*, a member of the thioredoxin family or another type of chaperone or redox catalyst might assist with the formation and cleavage of disulfide bonds between Cys59 and Cys155, and putatively additional linkages involving Cys21, Cys59 and Cys155.

A putative disulfide molecular switch modulating PR10 oligomerization based on BIA binding suggests the importance of BIA accumulation for protein–protein interactions in opium poppy latex and a role for the redox environment. This established the possibility of a dynamic environment in opium poppy latex linking oxidative state, BIA accumulation and protein–protein interactions.

## 5. Conclusions

We present crystallographic evidence of a putative disulfide molecular switch that is sensitive to the binding of BIA ligands in the opium poppy latex MLP/PR10 protein PR10-10. The novel crystal structures of the Cys59Ser and Cys155Ser mutants help to further define the role of the disulfide bond in stabilizing a ligand-free apo conformation that is disfavoured in both mutants. In combination with our previously published work on the structure and function of PR10-10 and other PR10 proteins in opium poppy latex, our data suggest for the first time that MLP/PR10 proteins may play key roles in the storage or transport of BIAs and other hydrophobic metabolites through the formation of protein bodies or biomolecular condensate-like aggregates in the latex of opium poppy and perhaps other metabolic compartments. BIA ligand binding to PR10-10 causes reduction of the intermolecular Cys59–Cys155 disulfide bond, subsequently changing the oligomeric state of PR10-10. In the context of latex protein bodies or biomolecular condensates, the oligomerization of PR10-10 may extend to other MLP/PR10 proteins or biosynthetic enzymes. These results contribute to the developing hypothesis that protein–protein interactions in the latex are mediated by BIA binding.

## Supplementary Material

PDB reference: PR10-10, C155S mutant, 8vo1

PDB reference: C59S mutant, 8vo2

PDB reference: C59S mutant, complex with papaverine, 8vo3

Supplementary Figures and Tables. DOI: 10.1107/S2059798324007733/jb5065sup1.pdf

## Figures and Tables

**Table 1:** Crystallographic statistics for apo and holo crystal structures of PR10-10-Cys59Ser and PR10-10-Cys155Ser

	PR10-10-Cys59Ser	PR10-10-Cys59Ser–papaverine	PR10-10-Cys155Ser
Data-collection statistics			
Space group	*C*222_1_	*C*222_1_	*C*222_1_
*a*, *b*, *c* (Å)	48.9, 71.1, 91.9	48.8, 71.5, 92.3	48.4, 70.8, 92.2
α, β, γ (°)	90, 90, 90	90, 90, 90	90, 90, 90
Wavelength (Å)	0.97946	0.97946	0.97946
Temperature (K)	100	100	100
Resolution (Å)	36.8–1.5 (1.54–1.50)	36.9–1.5 (1.54–1.50)	36.7–1.8 (1.84–1.80)
*R*_merge_	0.044 (1.015)	0.027 (0.888)	0.047 (1.252)
*R*_meas_	0.046 (1.104)	0.029 (0.961)	0.050 (1.316)
CC_1/2_	99.8 (77.3)	100.0 (80.8)	100.0 (80.2)
〈*I*/σ(*I*)〉	25.32 (1.76)	33.57 (1.90)	29.15 (1.90)
Completeness (%)	99.2 (93.6)	98.2 (85.2)	99.7 (99.1)
Multiplicity	10.3 (9.9)	10.3 (6.8)	10.9 (10.4)
Refinement			
Resolution (Å)	36.8–1.5	36.9–1.5	36.7–1.8
Unique reflections	25848	25857	15006
*R*_work_/*R*_free_	0.2030/0.2377	0.2088/0.2390	0.2035/0.2328
No. of atoms			
Total	1318	1303	1254
Protein	1181	1163	1167
Ligand	NA	25	NA
Water	137	115	87
Average *B* factors (Å^2^)			
Protein	29.7	35.0	38.3
Ligand	NA	50.5	NA
Water	36.6	41.2	42.2
R.m.s.d. from ideal geometry			
Bond lengths (Å)	0.006	0.009	0.008
Bond angles (Å)	0.86	1.14	1.02
Ramachandran outliers (%)	0.00	0.00	0.00
Ramachandran favored (%)	98.6	99.23	100.0
*MolProbity* score	0.93	1.24	1.28
Clashscore	1.72	4.70	5.22
PDB code	8vo2	8vo3	8vo1
