# Supplementary material for: Structural analysis of a ligand-triggered intermolecular disulfide switch in a major latex protein from opium poppy
Source: Acta Crystallogr D Struct Biol. 2024 Aug 29;80(Pt 9):675–85. doi: 10.1107/S2059798324007733 (PMC11394122; doi:10.1107/S2059798324007733)
Supplement: Supplementary file 1 [file d-80-00675-sup1.pdf]

STRUCTURAL BIOLOGY

Volume 80 (2024)

Supporting information for article:

Structural analysis of a ligand-triggered intermolecular disulfide switch in a major latex protein from opium poppy

Samuel C. Carr, Peter J. Facchini and Kenneth K. S. Ng

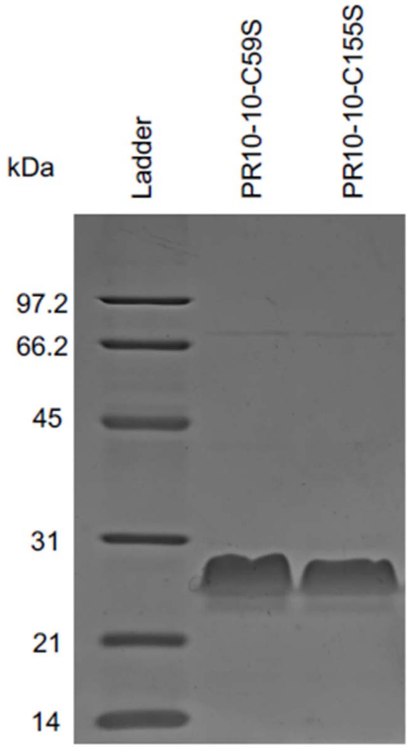

Figure S1 SDS-PAGE of recombinant purified PR10-10 cysteine mutants. Recombinantly expressed and cobalt-NTA affinity purified PR10-10 cysteine mutants. Proteins were purified using cobalt-NTA affinity chromatography and visualized on a 10% (w/v) polyacrylamide gel stained with Coomassie blue. The molecular weight marker is shown on the left side of the gel and the Cys59Ser and Cys155Ser mutants are labelled above their respective lane.

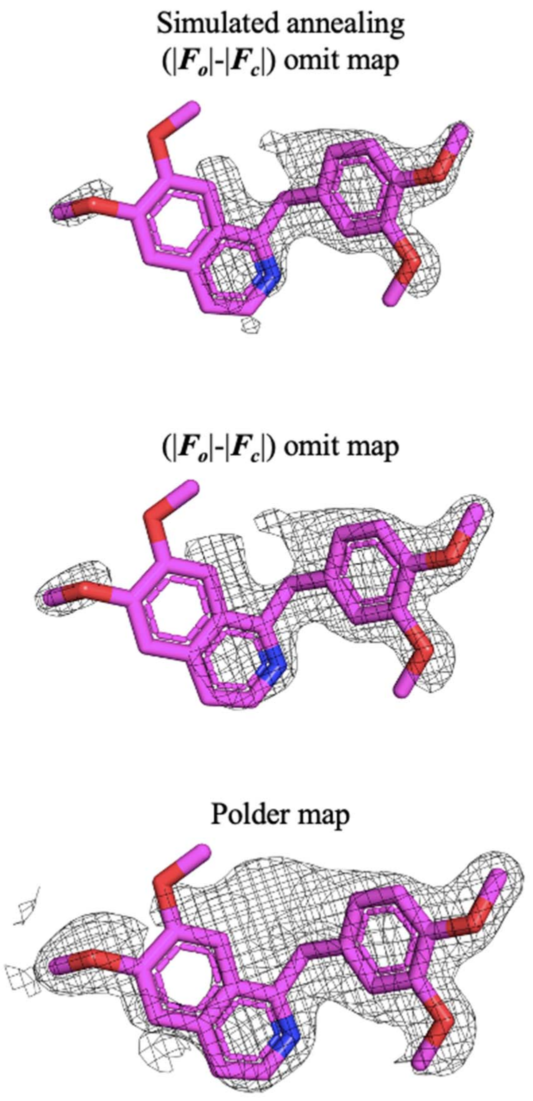

Figure S2 Comparisons between omit maps and polder map for the PR10-10-Cys59Ser complex with papaverine. Omit maps for the modeling of papaverine into PR10-10-C59S. All maps are calculated using PHENIX (Adams *et al.* 2010; Liebschner *et al.* 2017) contoured at 3σ and are represented as a grey mesh. The simulated annealing (|$\boldsymbol{F_o}$|-|$\boldsymbol{F_c}$|) omit map (from Fig. 4) used simulated annealing starting and ending temperatures of 5000K and 300K. Ligand carbon atoms and bonds are shown in magenta, oxygen in red, and nitrogen in blue.

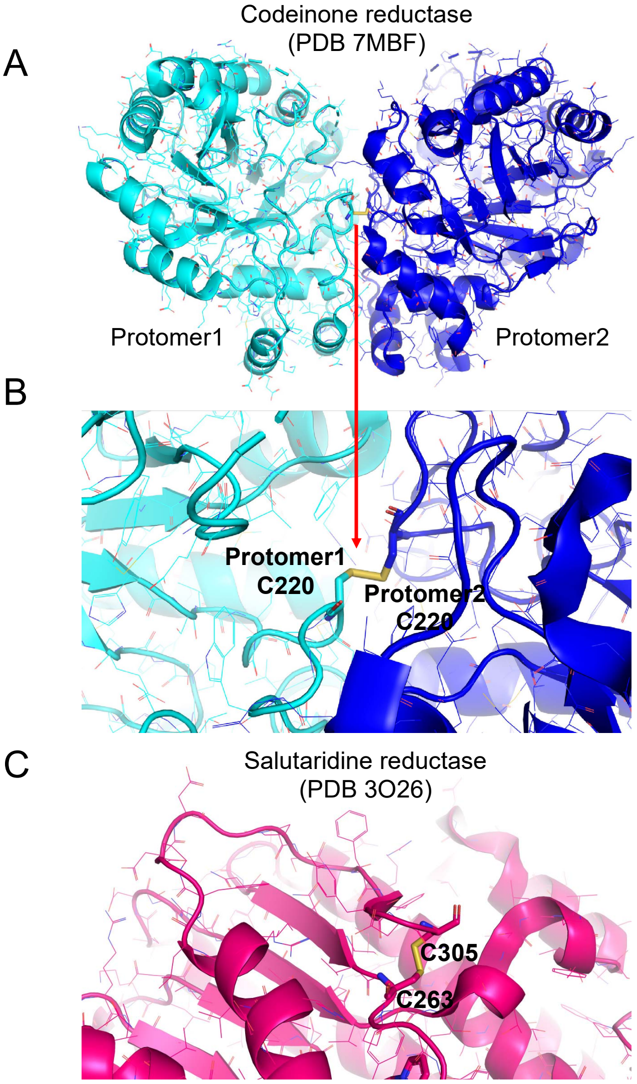

Figure S3 Disulfide bonds in codeinone reductase and salutaridine reductase. Disulfide bonds in *P. somniferum* BIA biosynthetic enzyme crystal structures. Carbon atoms and bonds are shown in cyan and blue for codeinone reductase, and magenta for salutaridine reductase. Nitrogen atoms are colored blue, oxygen atoms red, and sulfur atoms yellow. Disulfide bonds are shown in yellow. A) Codeinone reductase from the *apo* crystal structure (PDB 7MBF) (Carr *et al.* 2021). The homodimer seen in the crystal structure of codeinone reductase with protomers shown in cyan and blue. The disulfide bond linking both protomers is shown. B) Close up of the C120-C120 intermolecular disulfide bond between protomer loops forming part of the NADP(H) binding pocket. C) Salutaridine reductase from the *holo* crystal structure (PDB 3O26) (Higashi et al. 2011). The intramolecular disulfide bond formed between C305 and C263 stabilizes a weak interaction between and loop and a β-strand (Higashi *et al.* 2011).

Table S1 Primers used for *P. somniferum* PR10-10 mutagenesis

Primer Name	Sequence (5' to 3')	T_m (°C)
PR10-10-Cys59Ser-F	AGCGGTAGCATCAAAGAATGGAACTACGTTCTGGAG	75
PR10-10-Cys59Ser-R	GATGCTACCGCTCACCAGACCGTCACCCT	79
PR10-10-Cys155Ser-F	ACATCAGCTCCTCTGCGTAAGGATCCGGC	77
PR10-10-Cys155Ser-R	GAGGAGCTGATGTGGGCGGACATATCAACGAT	76
PR10-10-Cys21Ser-F	GAACAGCGACGCGGACATTTTCTATAAGATCGTTAAAC ATCA	74
PR10-10-Cys21Ser-R	CGTCGCTGTTCACTTCCAGTTGGGTAACCAG	76

Table S2 PR10-10 cysteine mutant nucleotide sequences

PR10-10-Cys59Ser	ATGGGCCATCATCATCATCATCATCATCATCATCACTCCGCGGCTCTTGAGGTGCTCTTT<br>CAGGGACCCATGGCTCATCACGGTGTTTCTGGTTTGGTGGGCAAGCTGGTTACCCAACT<br>GGAAGTGAACTGCGACGCGGACATTTTCTATAAGATCGTTAAACATCACGAAGAAGTTC<br>CGAATGTGATTCCGCATTTCTTTACCGGTGTTCAAGTGACCAAGGGTGACGGTCTGGTG<br>AGCGGTAGCATCAAAGAATGGAACTACGTTCGAGGGTAAAGCGATGACCGCTGTGGAA<br>GAAACCACCCATGCAGATGAAACCCGTACCCTGACCCATACATTACCGAAGGTGACGC<br>GATGAAGGACTACAAAAAGTTCGACGTTATCGTGGAAACCAATCCGAAGCGAACGGTC<br>ACGGTAGCGTTGTGACCTACAGCATCGTTTACGAAAAGATTAATGAAGATAGCCCGGC<br>GCCGTTCGACTACTTGAAATTCTTCCACCAAAACATCGTTGATATGTCCGCCCACATCTG<br>CTCCTCTGCGTAA
PR10-10-Cys155Ser	ATGGGCCATCATCATCATCATCATCATCATCATCACTCCGCGGCTCTTGAGGTGCTCTTT<br>CAGGGACCCATGGCTCATCACGGTGTTTCTGGTTTGGTGGGCAAGCTGGTTACCCAACT<br>GGAAGTGAACTGCGACGCGGACATTTTCTATAAGATCGTTAAACATCACGAAGAAGTTC<br>CGAATGTGATTCCGCATTTCTTTACCGGTGTTCAAGTGACCAAGGGTGACGGTCTGGTG<br>AGCGGTTGCATCAAAGAATGGAACTACGTTCTGGAGGGTAAAGCGATGACCGCTGTGG<br>AAGAAACCACCCATGCAGATGAAACCCGTACCCTGACCCATCACATTACCGAAGGTGA<br>CGCGATGAAGGACTACAAAAAGTTCGACGTTATCGTGGAAACCAATCCGAAGCCGAAC<br>GGTCACGGTAGCGTTGTGACCTACAGCATCGTTTACGAAAAGATTAATGAAGATAGCCC<br>GGCGCCGTTCGACTACTTGAAATTCTTCCACCAAAACATCGTTGATATGTCCGCCCACA<br>TCAGCTCCTCTGCGTAA
PR10-10-Cys21Ser	ATGGGCCATCATCATCATCATCATCATCATCATCACTCCGCGGCTCTTGAGGTGCTCTTT<br>CAGGGACCCATGGCTCATCACGGTGTTTCTGGTTTGGTGGGCAAGCTGGTTACCCAACT<br>GGAAGTGAACAGCGACGCGGACATTTTCTATAAGATCGTTAAACATCACGAAGAAGTT<br>CCGAATGTGATTCCGCATTTCTTTACCGGTGTTCAAGTGACCAAGGGTGACGGTCTGGT<br>GAGCGGTTGCATCAAAGAATGGAACTACGTTCTGGAGGGTAAAGCGATGACCGCTGTG<br>GAAGAAACCACCCATGCAGATGAAACCCGTACCCTGACCCATCACATTACCGAAGGTG<br>ACGCGATGAAGGACTACAAAAAGTTCGACGTTATCGTGGAAACCAATCCGAAGCCGAA<br>CGGTCACGGTAGCGTTGTGACCTACAGCATCGTTTACGAAAAGATTAATGAAGATAGCC<br>CGGCGCCGTTCGACTACTTGAAATTCTTCCACCAAAACATCGTTGATATGTCCGCCCAC<br>ATCTGCTCCTCTGCGTAA
PR10-10 wildtype	ATGGGCCATCATCATCATCATCATCATCATCATCACTCCGCGGCTCTTGAGGTGCTCTTT<br>CAGGGACCCATGGCTCATCACGGTGTTTCTGGTTTGGTGGGCAAGCTGGTTACCCAACT<br>GGAAGTGAACTGCGACGCGGACATTTTCTATAAGATCGTTAAACATCACGAAGAAGTTC<br>CGAATGTGATTCCGCATTTCTTTACCGGTGTTCAAGTGACCAAGGGTGACGGTCTGGTG<br>AGCGGTTGCATCAAAGAATGGAACTACGTTCTGGAGGGTAAAGCGATGACCGCTGTGG

	AAGAAACCACCCATGCAGATGAAACCCGTACCCTGACCCATCACATTACCGAAGGTGA<br>CGCGATGAAGGACTACAAAAAGTTCGACGTTATCGTGGAAACCAATCCGAAGCCGAAC<br>GGTCACGGTAGCGTTGTGACCTACAGCATCGTTTACGAAAAGATTAATGAAGATAGCCC<br>GGCGCCGTTCGACTACTTGAAATTCTTCCACCAAAACATCGTTGATATGTCCGCCCACA<br>TCTGCTCCTCTGCGTAA
